# Mind-Body Practice, Cardiovascular Health, and Well-Being: A 20-Years Study in US Adults

**DOI:** 10.1093/geroni/igaf122.2391

**Published:** 2025-12-31

**Authors:** Kallol Kumar Bhattacharyya, Sharmila Acharya

**Affiliations:** The University of Memphis, Memphis, Tennessee, United States; The University of Memphis, Memphis, Tennessee, United States

## Abstract

Mind-body practice is associated with various trajectories of health. However, the exact role of cardiovascular health in the association between mind-body practice and holistic well-being in mid and late life is still a growing field of research. We examined participants enrolled in waves 1-3 (1995–2015) of the Midlife in the United States (MIDUS) study (N = 2,536) and merged data from the main self-administered questionnaire (SAQ) and biomarker projects. Holistic well-being was assessed by flourishing score, a composite construct of emotional, psychological, and social well-being. We used structural equation models to examine whether persistent mind-body practice across two waves (1–2) or intermittent practice at one wave is associated with better flourishing over 20 years, compared to no practice, while controlling for prior wave flourishing and covariates (baseline socio-demographics, health, and functional status). Additionally, we assessed if ideal cardiovascular health, which was measured using the parameters of Life’s Essential 8 defined by the American Heart Association, mediates the above associations. Findings suggested that persistent mind-body practice has a significant positive effect (b = 1.052; SE = 0.531; p < .05) on flourishing. Ideal cardiovascular health status also has a significant positive effect (b = 0.303; SE = 0.141; p < .05) on flourishing; however, it does not mediate the association between persistent mind-body practice and flourishing. More research is necessary to support future policy and practice recommendations validating the current findings to enhance the well-being of more vulnerable populations, such as long-term care residents.

